# An Efficiently Cleaved HIV-1 Clade C Env Selectively Binds to Neutralizing Antibodies

**DOI:** 10.1371/journal.pone.0122443

**Published:** 2015-03-30

**Authors:** Saikat Boliar, Supratik Das, Manish Bansal, Brihaspati N. Shukla, Shilpa Patil, Tripti Shrivastava, Sweety Samal, Sandeep Goswami, C. Richter King, Jayanta Bhattacharya, Bimal K. Chakrabarti

**Affiliations:** 1 THSTI-IAVI HIV Vaccine Design Program, Translational Health Science and Technology Institute, 496 Udyog Vihar, Phase-III, Gurgaon-122 016, Haryana, India; 2 IAVI Vaccine Design, New York, NY, United States of America; CIP, NCI-Frederick, NIH, UNITED STATES

## Abstract

An ideal HIV-1 Env immunogen is expected to mimic the native trimeric conformation for inducing broadly neutralizing antibody responses. The native conformation is dependent on efficient cleavage of HIV-1 Env. The clade B isolate, JRFL Env is efficiently cleaved when expressed on the cell surface. Here, for the first time, we report the identification of a native clade C Env, 4-2.J41 that is naturally and efficiently cleaved on the cell surface as confirmed by its biochemical and antigenic characteristics. In addition to binding to several conformation-dependent neutralizing antibodies, 4-2.J41 Env binds efficiently to the cleavage-dependent antibody PGT151; thus validating its native cleaved conformation. In contrast, 4-2.J41 Env occludes non-neutralizing epitopes. The cytoplasmic-tail of 4-2.J41 Env plays an important role in maintaining its conformation. Furthermore, codon optimization of 4-2.J41 Env sequence significantly increases its expression while retaining its native conformation. Since clade C of HIV-1 is the prevalent subtype, identification and characterization of this efficiently cleaved Env would provide a platform for rational immunogen design.

## Introduction

The envelope glycoprotein (Env) of human immunodeficiency virus type-1 (HIV-1) is a surface molecule that mediates virion entry into target cells. It undergoes conformational changes following engagement of the primary receptor, CD4 and subsequently binds to the co-receptor, which ultimately leads to virus-cell membrane fusion and entry of the virus into target cells [1–12]. Due to its essential role in viral infectivity, Env is the primary target of neutralizing antibodies (nAb) and therefore is the focus of global HIV-1 vaccine immunogen design efforts.

In natural HIV-1 infections, approximately 10–20% of chronically infected individuals develop “neutralization breadth”. Serum antibodies from these individuals can potently neutralize an array of HIV-1 isolates of different clades. This broad neutralizing activity, generated during years of natural infection, is mediated by monoclonal antibodies whose target epitopes have been mapped to various sub-regions of the Env such as the CD4 binding site (CD4-bs), various glycans and the membrane proximal external region (MPER) [13–21]. Many of these broadly neutralizing antibodies (bnAb) are dependent on Env conformation and the recently isolated bnAb, PGT151 is also cleavage specific [22, 23]. In the absence of anti-retroviral therapy, passive immunization with a combination of bnAbs can suppress viremia and is protective *in vivo*, suggesting their potential effectiveness if they could be elicited through a vaccine [24–33].

Efforts to develop an HIV-1 vaccine have been challenging, although recent studies suggest some promising approaches. One strategy for a highly effective vaccine is to design an immunogen that could elicit bnAbs similar to those seen in infected individuals. An essential component of designing such an immunogen is conservation of the native trimeric structure of Env. A functional Env spike is formed by cleavage of gp160 glycoprotein by the furin protease into two subunits: the exterior gp120 and the transmembrane gp41. The cleaved oligomeric subunits of Env protein remain non-covalently bound to form the mature, meta-stable Env structure. Efficient cleavage of Env plays an important role in imparting its native trimeric conformation [34]. It has also been shown that native-like trimers of HIV-1 Env in its properly cleaved form, either as a soluble or cell surface expressed protein, binds exclusively to broadly neutralizing antibodies but the uncleaved form does not discriminate between neutralizing and non-neutralizing antibodies [35–37]. An effective system for presenting HIV-1 Env as a membrane-anchored immunogen is through a genetic vaccine method such as a virus vector or plasmid DNA. Since it would be important for an HIV-1 Env vaccine candidate to bear structural resemblance and demonstrate similar physio-chemical properties as the Env present on infectious viruses, expression of an efficiently cleaved Env in its native trimeric conformation on the cell surface through genetic vaccination could prompt the immune system towards elicitation of potent neutralizing antibodies.

To date, only a clade B primary isolate, JRFL has been shown to be naturally and efficiently cleaved when expressed on the cell surface [35, 36]. A modified version of soluble, trimeric BG505 SOSIP.664 Env also demonstrates efficient cleavage when transiently expressed in mammalian cells in the presence of exogenous furin [38]. Prior studies have shown that priming with plasmid DNA expressing cleaved JRFL Env and boosting with protein in nonhuman primates results in induction of a robust nAb response of limited breadth [39]. A reasonable approach to expand the breadth of this nAb response would be through simultaneous immunization with naturally cleaved Envs from other clades. In fact, viral diversity and mutations probably favor development of neutralization breadth as chronically and super-infected individuals tend to develop bnAb response more often [40–42]. This implies that a combination of multiple cleavage competent Envs with diverse genetic background in a vaccine may increase the likelihood of a broadly reactive antibody response. Thus, identification of an efficiently cleaved Env from subtype C, which is responsible for nearly half of the HIV-1 infections globally, would significantly contribute towards designing an HIV-1 vaccine that can elicit broad and potent nAb response.

In this study, we report for the first time the identification of a cleavage competent clade C HIV-1 Env, 4-2.J41, from the plasma of an HIV infected individual in India. This Env efficiently exposes epitopes for an array of known broadly neutralizing antibodies. Our results indicate that 4-2.J41, when expressed on the cell surface, likely forms a native conformation. We also demonstrate that the codon-optimized 4-2.J41 Env, unlike JRFL, displays similar properties as its non-codon optimized counterpart. These findings have important implications in HIV-1 vaccine research as 4-2.J41 can provide an additional platform for Env based immunogen design.

## Results

### Screening and identification of an efficiently cleaved Indian subtype C HIV-1 Env

**Fig 1:**
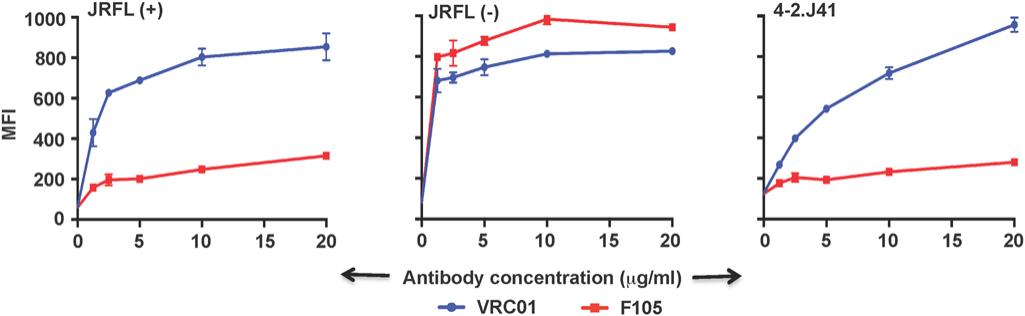
Selection of HIV-1 Envs for cleavage by cell surface Ab binding assay. Representative FACS-based cell surface staining curves with Mean Fluorescent Intensity (MFI) values of cleavage-competent JRFL Env, JRFL(+), (left panel) cleavage-defective JRFL Env, JRFL(-) (middle panel) and 4-2.J41 (right panel) expressed on 293T cells with CD4-bs neutralizing antibody, VRC01 and non-neutralizing antibody, F105. Bars at each antibody concentration indicate the SEM values for duplicate samples. The JRFL(+) and JRFL(-) Env clones are cytoplasmic-tail truncated at a.a. 709, unless mentioned otherwise.

In order to screen for naturally cleaved Env sequences derived from clade C viruses found in Indian individuals, a total of 38 Env clones were examined by transient transfection of 293T cells. Ten Env clones were selected from preliminary screening of Env transfected cell-lysate by Western blot for signs of cleavage i.e. presence of both gp120 and gp160 proteins in the gel (data not shown). The selected 10 Env clones were further investigated for their cleavage efficiency by FACS based cell surface antibody binding assay, as described in materials and methods. The Envs tested were found to bind the neutralizing and non-neutralizing antibodies at varying concentrations. Based on the ratio of binding (Mean Fluorescent Intensity: MFI) to CD4-bs directed neutralizing vs. non-neutralizing antibody (VRC01 vs. F105) (S1 Table), which is indicative of the degree of cleavage, the Env clone 4-2.J41 was determined to be the most efficiently cleaved when expressed on the cell surface. In Fig. 1, representative graphs show binding of CD4-bs directed neutralizing antibody, VRC01 and CD4-bs directed non-neutralizing antibody, F105 to cleaved and uncleaved Envs. As reported earlier [39], the cleavage competent JRFL Env, JRFL (+) bound efficiently to neutralizing antibody VRC01 but weakly to non-neutralizing antibody F105 (Fig. 1 left panel), whereas the cleavage-defective JRFL Env, JR-FL (-) Env, as expected, bound both neutralizing and non-neutralizing antibodies efficiently (Fig. 1 middle panel). Similar to JRFL (+) Env, the newly identified clade C Env, 4-2.J41 bound efficiently to the neutralizing antibody and weakly to the non-neutralizing antibody, which indicates efficient cleavage of this Env (Fig. 1 right panel). Subsequently, additional evaluations and characterizations were performed with the selected Env, 4-2.J41.

### Env 4-2.J41 is efficiently cleaved on the cell surface

**Fig 2:**
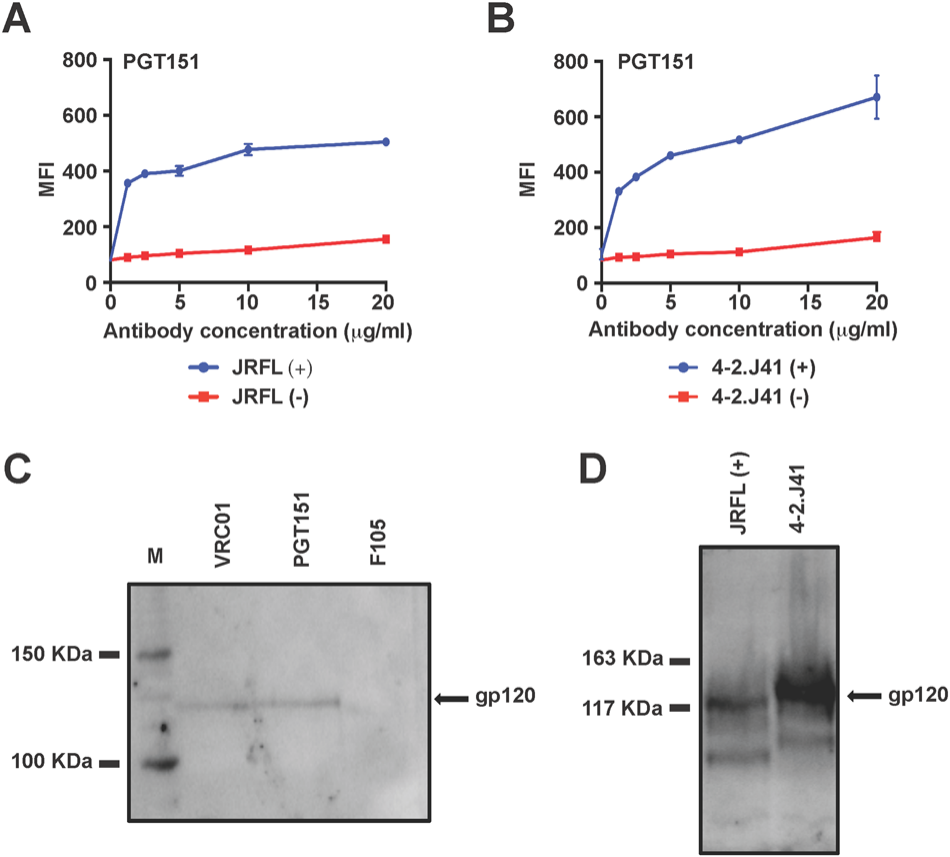
Efficient cleavage of 4-2.J41 Env on the cell surface. (A-B) FACS-based cell surface binding curves of wild type and cleavage-defective JRFL (A) and 4-2.J41 (B) Env to cleavage specific antibody PGT151. The wild type and cleavage-defective Envs are designated by (+) and (-), respectively. Mean fluorescence intensities (MFI) of antibody binding are shown. The graphs shown here are derived from the same representative experiments. Bars at each antibody concentration indicate the SEM values for duplicate samples. (C) Western blot analysis of immunoprecipitated 4-2.J41 Env protein from plasma membrane fraction. Proteins from the plasma membrane fraction of Env transfected 293T cells were immunoprecipitated with VRC01, PGT151 and F105 antibodies and analyzed by Western blot using HIVIG as probe. M = molecular weight marker. (D) Western blot analysis of Env glycoproteins from cell surface biotinylation. Cell surface expressed JRFL(+) and 4-2.J41 Envs were biotinylated, lysed and immunoprecipitated with neutravidin-agarose before analysis by Western blot and probed with HIVIG.

To further confirm the cleavage-competency of 4-2.J41 Env when expressed on the cell surface, its binding ability to a cleavage-dependent neutralizing antibody, PGT151 was determined. This recently isolated antibody demonstrates selective binding to properly cleaved Env proteins [22, 23]. Fig. 2A confirms this finding as PGT151 recognized JRFL (+) Env efficiently, but JRFL (-) Env binds to this antibody only very weakly. Similarly, PGT151 antibody bound efficiently to the wild-type 4-2.J41 Env and this binding was significantly reduced in the cleavage-defective 4-2.J41 Env (Fig. 2B).

In order to determine the proportion of cleaved Envs on the cell surface, we compared binding of 4-2.J41 Env to cleavage-dependent antibody PGT151 in relation to a non-cleavage-dependent antibody VRC01 and non-neutralizing antibody F105. This was confirmed biochemically using purified plasma membrane fraction of the Env expressing 293T cells (Fig. 2C). Equal amount of purified membrane fraction containing 4-2.J41 Env was immunoprecipitated with 2μg of PGT151, VRC01 and F105 antibodies, separated in SDS-PAGE followed by Western blot using Env-specific antibody. The intensity of bands migrating at identical position of gp120 in both VRC01 and PGT151 lanes were found to be similar indicating that equal amount of gp120 was pulled down by VRC01 and PGT151 antibodies (Fig. 2C). In contrast, no or a very low intensity band was observed with F105 antibody under identical condition (Fig. 2C). These data in Fig. 2C suggest that majority of 4-2.J41 Envs expressed on the cell surface are in cleaved and native trimeric conformation. These results were verified again with an alternate biochemical approach. The Env expressing viable 293T cells were labeled with biotin as described in the materials and methods section. It is to be noted that the biotin-labeling reagent is impermeable to live cells. The biotinylated cells were lysed and the proteins in cell lysate were precipitated with neutravidin-agarose. The pulled proteins were analyzed in SDS-PAGE followed by Western blot. We observed only a band migrating at the position of gp120 protein and no band migrating at position of gp160 was detected (Fig. 2D). These data also confirms the presence of primarily a cleaved form of 4-2.J41 Env on the cell surface.

These FACS and protein-gel data cumulatively provide evidence for efficient cleavage of the Indian clade C Env, 4-2.J41 on the cell surface and majority of the cell surface expressed 4-2.J41 Envs are present in a cleaved, native and trimeric conformation.

### Characterization of bnAb epitopes and conformation of cell surface expressed 4-2.J41 Env

**Fig 3:**
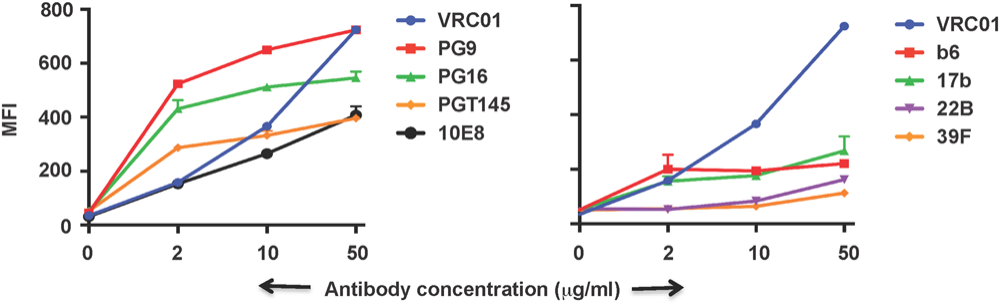
Neutralizing and non-neutralizing antibody binding curves of 4-2.J41 Env. Mean fluorescence intensities (MFI) of FACS-based binding assay for neutralizing (VRC01, 10E8, PG9, PG16, PGT145) and non-neutralizing (b6, 17b, 39F, 22B) antibodies are shown. The graphs shown here are derived from the same representative experiments. Bars at each antibody concentration indicate the SEM values for duplicate samples.

Recent discovery and characterization of several bnAbs that bind to distinct and well defined epitopes and the requirement of some of these antibodies for a particular conformation of the Env protein allowed us to probe the presence of bnAb epitopes and native conformation of cell surface expressed 4-2.J41 Env. To characterize its antigenic properties, we assessed binding of 4-2.J41 Env to set of neutralizing and non-neutralizing antibodies. The MFIs of antibody binding were determined over a range of concentrations. We tested antibodies, which bind to one of four well-defined epitopes on HIV-1 Env: the CD4-bs, the trimer selective V2 “cap”, the V3 region and the MPER region. Env 4-2.J41 bound efficiently to CD4-bs-directed neutralizing antibody VRC01 in comparison to non-neutralizing antibodies F105 and b6 (Fig. 3). For the V2 region, 4-2.J41 Env bound to trimer conformation-dependent antibodies PG9, PG16 and PGT145 (Fig. 3). Similarly, 4-2.J41 Env was also recognized by the V3 (PGT121) and MPER (10E8) targeted antibodies tested, indicating presence of their respective epitopes on this Env (Fig. 3, S2 Fig.). In contrast, 4-2.J41 showed minimal binding to the non-neutralizing antibodies tested (Fig. 3). These binding data is also in congruence with the neutralization results where viruses pseudotyped with 4-2.J41 Env were tested for neutralization sensitivity with a panel of antibodies (S2 Table). These findings provide further evidence of efficient cleavage of the Indian clade C Env, 4-2.J41 as only epitopes for broadly neutralizing antibodies but not non-neutralizing antibodies are exposed. Most importantly, binding of PG16 and PGT145 antibodies to this Env also suggests that 4-2.J41 Env likely assumes a native trimeric conformation when expressed on the cell surface.

### Cleavage-defective mutations alter conformation of 4-2.J41 Env

**Fig 4:**
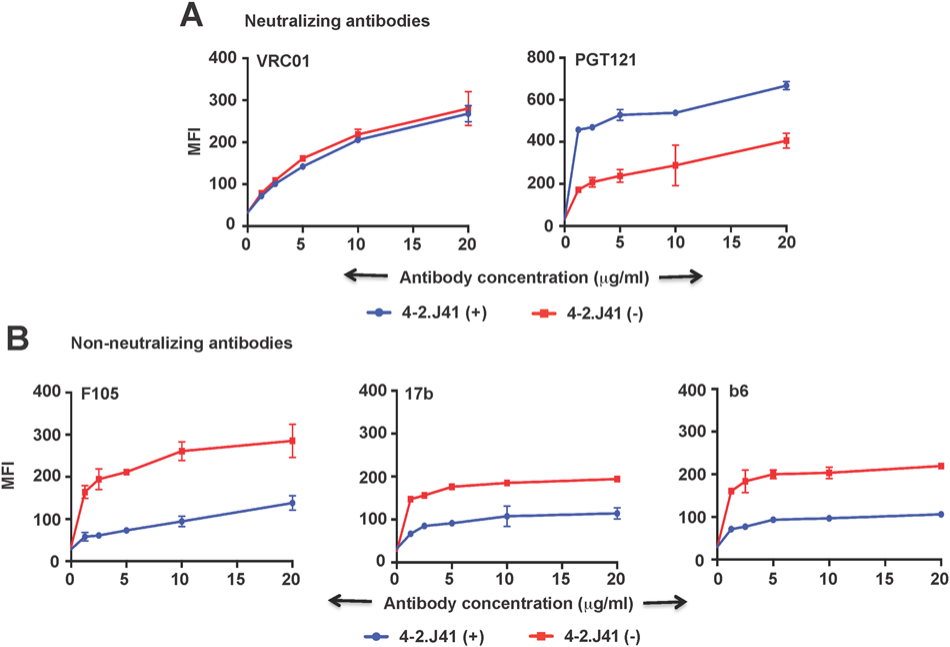
Effects of cleavage-defective mutations in 4-2.J41 Env to antibody binding. The wild type and cleavage-defective Envs are designated by (+) and (-), respectively. Mean fluorescence intensities (MFI) of binding of neutralizing (VRC01, PGT121) and non-neutralizing antibodies (F105, 17b, b6) are shown in panel (A) and (B), respectively. The graphs shown here are derived from the same representative experiments. Bars at each antibody concentration indicate the SEM values for duplicate samples.

Next, we examined whether inability of non-neutralizing antibodies to efficiently bind 4-2.J41 Env was due to structural occlusion or absence of their respective epitopes in this Env. To test this, the cleavage recognition site of furin in the Env was mutated (REKR to SEKS) to render this Env cleavage-defective. When assessed for their binding efficiency to different antibodies, it was found that the cleavage defective mutant of 4-2.J41 Env retained similar binding properties to the CD4-bs directed non-cleavage specific neutralizing antibody, VRC01 (Fig. 4A). However, recognition by conformation dependent neutralizing antibody, PGT121 was reduced in the cleavage-defective mutant (Fig. 4A). In addition, binding to non-neutralizing antibodies directed to either CD4 or co-receptor binding site was significantly increased (Fig. 4B). This indicates that although 4-2.J41 Env has the required epitopes, the non-neutralizing antibodies bind inefficiently to this Env probably because of steric hindrance that keeps the epitopes for non-neutralizing antibodies unexposed. Decreased binding of conformational antibodies to cleavage defective 4-2.J41 Env also indicates significant conformational differences between cleavage-efficient and cleavage-defective Envs.

### Soluble CD4 induces shedding of 4-2.J41 gp120 from cell surface

**Fig 5:**
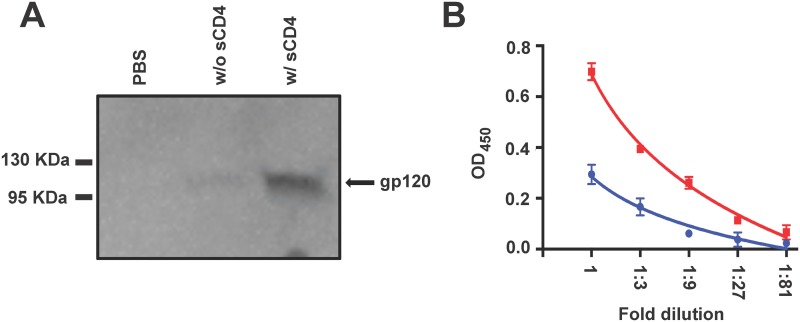
CD4-induced shedding of gp120 from 4-2.J41 Env spikes. (A) Equal number of 4-2.J41 Env expressing 293T cells was incubated with or without 40g of sCD4 for 1 hour. Cell supernatant was harvested and analyzed by Western blot using anti-gp120 clade C rabbit polyclonal antibody as probe. (B) Equal number of 4-2.J41 Env expressing 293T cells were treated with or without 50g of sCD4 for 1 hour at 4°C and the cell supernatants were analyzed by ELISA for the presence of “shed” gp120. The gp120 present in cell supernatants were captured on ELISA plates by pre-coating with lectin and then detected with anti-gp120 clade C rabbit polyclonal antibody. Bars at each dilution indicate the SEM values for duplicate samples.

The two sub-units of an efficiently cleaved Env, gp120 and gp41 remain non-covalently attached to form a meta-stable hetero-dimer, which tends to undergo some level of self-dissociation in solution known as spontaneous shedding. Engagement of soluble CD4 (sCD4) brings about conformational changes leading to significant increase in the release of the extracellular gp120 component, often termed as induced shedding. To assess the relative stability in solution and sCD4 dependent conformation changes, we characterized both spontaneous and induced shedding of the 4-2.J41 Env. Equal numbers of 293T cells expressing Env proteins were incubated in the absence or presence of sCD4 and the gp120 protein in the culture supernatant was measured to assess spontaneous vs. induced shedding, respectively. The measurement of gp120 in the supernatant was done using two different methods, namely Western blot and ELISA. In the first method, gp120 proteins present in the supernatant of cells incubated with or without sCD4 were analyzed directly by Western blot (Fig. 5A). Without sCD4 treatment, there was minimal amount of spontaneous gp120 shedding while sCD4 treatment induced significant increase in gp120 shedding (Fig. 5A). Similar result of almost three-fold increase in gp120 shedding following sCD4 treatment was obtained when gp120 in the supernatant was measured using an ELISA based assay (Fig. 5B). This indicates that considerable amount of Env proteins remain in gp120-gp41 meta-stable formations before sCD4 treatment and sCD4 treatment induces conformational changes leading to increased shedding. Additionally, when sCD4 treated or untreated 293T cells expressing 4-2.J41 Env were stained with gp120 and gp41-targeted antibodies, binding of gp120-targeted antibodies (VRC01 and PGT121) decreased following sCD4 treatment whereas recognition by gp41-targeted antibody 7B2 increased, clearly indicating enhanced exposure of the gp41 stalk due to shedding of gp120 from the cell surface (S1 Fig.). These data provided further evidence of efficient cleavage of 4-2.J41 Env when expressed on the cell surface and changes in conformation following exposure to sCD4.

### Codon-optimized 4-2.J41 Env is efficiently cleaved and maintains its conformation

**Fig 6:**
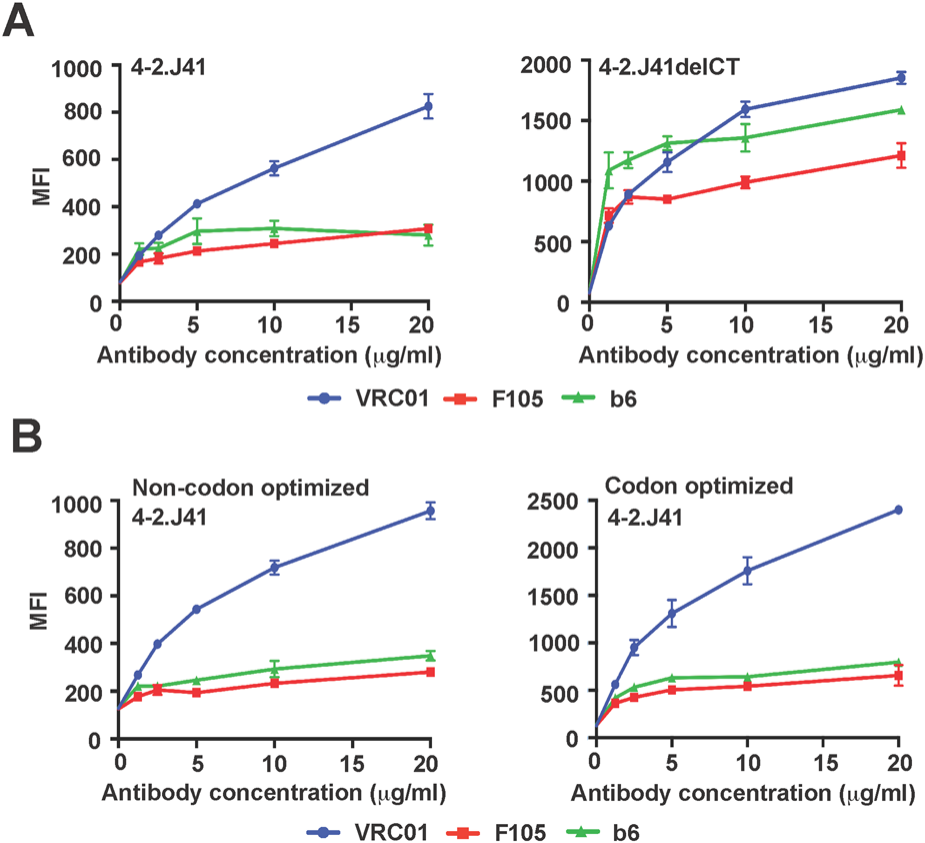
Effects of increased cell surface expression of 4-2.J41 Env to antibody binding. (A) Binding curves of VRC01, F105 and b6 antibodies to wild-type and cytoplasmic tail deleted 4-2.J41delCT Env. (B) Binding curves of VRC01, F105 and b6 antibodies to wild-type and codon-optimized 4-2.J41 Env. The graphs shown here are derived from the same representative experiments. Bars at each antibody concentration indicate the SEM values for duplicate samples.

We next investigated whether increased expression of 4-2.J41 Env, which is a desirable property of an immunogen, could affect its cleavage efficiency. Prior studies have documented that there is an increase in expression and incorporation of Env glycoprotein on the cell surface when the cytoplasmic tail of an Env is truncated before the conserved endocytosis motif [43]. Analysis of the tail-truncated mutant of 4-2.J41 Env revealed that there was an increase in expression compared to the full-length counterpart as evidenced from the increase in binding (MFI) of VRC01 (Fig. 6A) and PGT121 antibodies (S2A Fig.). However, when tested with non-neutralizing antibodies, recognition of tail-truncated 4-2.J41delCT Env by F105 and b6 was also significantly increased indicating a change in conformation (Fig. 6A). These findings are in contrast with observations reported for JRFL Env, where tail-truncation does not affect cleavage efficiency. Therefore, we took an alternative approach to increase 4-2.J41 Env expression by codon-optimization for mammalian expression under the robust CMV promoter. This codon-optimized 4-2.J41 Env showed a significant increase in expression on the cell surface in comparison to its wild-type homologue (Fig. 6B and S2B Fig.). However, this increased expression did not affect the conformation or cleavage competency of codon-optimized 4-2.J41 Env, as its binding properties to neutralizing and non-neutralizing antibodies remained unchanged (Fig. 6B). This data shows that codon-optimization of the Indian clade C Env, 4-2.J41, augments its expression on the cell surface without altering its native cleaved conformation.

## Discussion

An essential component of HIV-1 vaccine design is to mimic the native Env spikes on the virion in the immunogen. However, a great majority of HIV-1 Envs, when expressed on the cell surface, do not cleave properly and therefore, fail to assume the native structure. In the present study, we have identified a clade C primary isolate Env, 4-2.J41, which when expressed on the cell surface is efficiently cleaved and likely conform to the native structure as evidenced from its binding properties to several neutralizing and non-neutralizing antibodies. In this study, we used three methods to determine the cleavage competency of 4-2.J41. First, we used the ratio of binding of neutralizing (VRC01) and non-neutralizing (F105) antibodies directed to the CD4-bs to confirm that the non-neutralizing sites are occluded on cell surface expressed 4-2.J41 Env. This is a well established property for a native configuration, efficiently cleaved Env trimer such as JRFL [35]. It further suggests that CD4-bs of 4-2.J41 Env cell surface trimer is structurally similar to that of JRFL. Secondly, we showed that the cell surface expressed 4-2.J41 is recognized by PGT151, a recently isolated neutralizing antibody, which exclusively binds to envelope glycoproteins that have a native, cleaved conformation [22, 23]. When 4-2.J41 Env was rendered cleavage-defective by mutating the cleavage site, PGT151 failed to efficiently bind the mutant Env, further supporting our earlier observation that 4-2.J41 presents a cleaved native structure that can be recognized by PGT151 antibody. And lastly, biochemical analysis of the Env glycoprotein also demonstrated that 4-2.J41 Env expressed on the cell surface is mostly cleaved, as only a gp120 protein species was detected in cell membrane biotinylated samples. Immunoprecipitation of plasma membrane bound 4-2.J41 Env with VRC01, PGT151 and F105 antibodies further showed that majority of cell surface expressed Envs are in fact in a cleaved conformation as only neutralizing antibodies efficiently pulled-down membrane associated Env proteins. Results of the neutralization assays as well as cell surface binding assays confirmed that 4-2.J41 Env glycoproteins were recognized more efficiently by neutralizing antibodies as compared to non-neutralizing antibodies on virion as well as cell surface. This antigenic attribute of 4-2.J41 Env is desirable for designing immunogen to elicit bnAb response. The ability of 4-2.J41 Env to bind PG9, PG16 and PGT145 antibodies, which recognize quaternary epitopes in V2 domain of trimeric Env, suggests that 4-2.J41 also assumes native conformation when expressed on the cell surface. Additionally, since JRFL lacks the complete epitopes for PG9 and PG16 antibodies, this newly identified Env, 4-2.J41, could provide a better platform for immunogen design by exposing epitopes for a greater number of broadly neutralizing antibodies.

One desirable property for a genetically delivered immunogen is a high level of expression. We have demonstrated two methods to enhance expression of HIV-1 Env from plasmid DNA. Similar to JRFL, truncation of the cytoplasmic tail of 4-2.J41 resulted in increased expression on the cell surface. However, it also led to conformational changes as evidenced from binding of non-neutralizing antibodies F105 and b6 to 4-2.J41delCT Env. In marked contrast to the phenotype observed with tail-truncated JRFL Env, HIV-1 viruses pseudotyped with tail-truncated 4-2.J41 mutants became less sensitive to several neutralizing antibodies (S2 Table). Thus, unlike JRFL, the cytoplasmic tail of 4-2.J41 Env plays a significant role in maintaining the cleavage competency and native conformation of this Env. These results are in congruence with published reports where truncation of the cytoplasmic tail has been shown to alter conformation as well as epitope exposure of the Env ectodomain [44, 45]. We used codon-optimization of the *env* sequence as an alternate approach to increase expression of 4-2.J41 Env. As expected, codon-optimized 4-2.J41 showed an increase in expression on the cell surface. Interestingly, such modification in JRFL Env has been reported to result in a reduction in the level of cleavage [39]. However, in the case of 4-2.J41, codon-optimization did not compromise the cleavage efficiency of this Env. In addition to retention of cleavage efficiency, codon-optimization is advantageous over Env cytoplasmic tail-truncation as a method for increasing its expression as this system allows for use of a single plasmid for delivery of the immunogen.

In summary, in this study we have identified and characterized a clade C Env, 4-2.J41, which is naturally and efficiently cleaved on the cell surface. This Env selectively exposes epitopes for only neutralizing antibodies and presents native trimeric conformation in the membrane-anchored form. It is now apparent that a trimeric Env protein that is fully cleaved and assumes a native conformation would be a better immunogen for eliciting neutralizing antibodies. However, JRFL was the only naturally cleaved Env available on which such immunogens could be designed. Recent characterizations of a modified, soluble form of BG505 Env trimer have further bolstered the importance of a cleavage-competent Env in immunogen design. One obvious yet important implication of this study is that future structural and immunological analysis of 4-2.J41 Env would provide opportunities to validate the information available as well as gain new insights on naturally cleaved Envs that is so far restricted to one Env, JRFL. From a vaccine perspective, since clade C of HIV-1 virus is responsible for almost half of global infections, antibodies induced by this clade C Env could be effective against a larger pool of infecting viruses. Additionally 4-2.J41 Env was isolated from a recently infected individual and therefore could be more relevant as a vaccine immunogen against infecting or early-transmitted viruses [46]. This newly identified Env is efficiently cleaved in a membrane-bound form and therefore could serve as an important component in a DNA prime-protein boost vaccine regimen. Since 4-2.J41 Env is naturally cleaved, this full-length Env may prove to be an expedient platform for further development into a stable, soluble native trimeric protein. However, this remains to be investigated. Finally, in combination with JRFL, 4-2.J41 Env provides a promising avenue of immunization with multiclade cleaved Env immunogens. Such multiclade Env vaccination strategy has shown to increase neutralization breadth [47]. Therefore, identification of this cleavage-competent Env, 4-2.J41, from subtype C, will serve as an additional platform and broaden the approaches of designing potential immunogens for development of an effective HIV-1 vaccine.

## Materials and Methods

### Indian clade C HIV-1 Env, cell lines and antibodies

The full-length Env (gp160) clones were obtained from National AIDS Research Institute, Pune, India and have been described previously [48]. Tzm-bl (NIH AIDS Reagent Program) and 293T cells (ATCC) were maintained in Dulbecco’s modified Eagle medium (DMEM) containing 10% heat inactivated fetal bovine serum (HIFBS), 20 mM L-glutamine, 100 U/ml penicillin, and 100 μg/ml streptomycin. Anti-HIV-1 broadly neutralizing antibodies (VRC01, PGT121, PGT145, PGT151, PG9, PG16, 4E10, 10E8), non-neutralizing antibodies (F105, b6, 22B, 7B2, 17B, 39F) and HIVIG antibodies were obtained from the IAVI Neutralizing Antibody Center (TSRI, La Jolla, California). The anti-gp120 (clade C) rabbit polyclonal antibody was purchased from Immunetech, USA.

### FACS-based Env cell surface expression assay

Expression of HIV-1 Envs on the cell surface was assessed by flow cytometry as previously described [35, 36]. Briefly, 293T cells were transiently transfected with an Env-encoding pSVIII-plasmid in combination with a tat-expressing plasmid (env: tat = 20:1) using FuGENE 6 (Promega Inc). Forty-eight hours post-transfection, cells were harvested, washed thrice with FACS buffer (PBS with 5% FBS) and stained with different dilutions of monoclonal neutralizing and non-neutralizing antibodies for 1 hour. The cells were washed thrice and then stained with a PE-conjugated goat anti-human secondary antibody (1:200 dilution, Jackson ImmunoResearch) for 1 hour. Finally the cells were washed and resuspended in 0.5% paraformaldehyde (in PBS) for analysis with a FACS Verse/Canto analyzer (BD Bioscience). The data was analyzed with FlowJo software (version 10.0.6, Tree Star Inc).

### Plasma membrane-fraction isolation, biotinylation and Western blot assay

Plasma membrane fraction of Env-transfected 293T cells was isolated using the Plasma Membrane Protein Extraction Kit (Abcam) following manufacturer’s protocol. Briefly, 36–48 hours post-transfection, 293T cells were harvested with 0.5mM EDTA in PBS and then washed thrice with ice-cold PBS. Plasma membrane fraction was suspended in lysis buffer (10 mM Tris-HCl pH 8, 150 mM NaCl, 1 mM DTT, 1% Triton-X and protease inhibitors) and incubated on ice with repeated vortexing to isolate the plasma membrane proteins. The amount of protein extracted was quantified with Bradford’s reagent (BioRad). For immunoprecipitation, 15–20 μg of protein was incubated with 2 μg each of VRC01, PGT151 or F105 antibodies overnight at 4°C, followed by incubation with 60 μl slurry of Protein G agarose for 1 hour. Beads were washed thrice and analyzed by Western blot using purified IgG from pooled anti-HIV human polysera (HIVIG) as probes.

Biotinylation of cell surface expressed Env was carried out using Pierce Cell Surface Protein Isolation kit (Thermo Scientific). The cells were lysed and biotin-labeled proteins were pulled-down with NeutrAvidin agarose according to the manufacturer’s instructions for further analysis by Western blot.

For Western blot, 293T cells were harvested, washed once with PBS and then lysed by either directly heating at 100°C in 1X SDS-sample loading buffer or by suspending in lysis buffer. In the case of lysis buffer, cells were sonicated, incubated on ice with intermittent mixing for 30 minutes and then centrifuged at 13000 rpm and 4°C for 30 minutes. The supernatant was collected and proteins in the supernatant were then precipitated on ice with 20% trichloroacetic acid for 30 minutes, followed by centrifugation at 13000 rpm and 4°C for 15 minutes, washing with acetone and air-drying. The precipitate was finally resuspended in 1X SDS-sample loading buffer by heating at 100°C. The samples were then analyzed for protein expression and cleavage by Western blot analysis using HIVIG as probes.

### Analysis of spontaneous and sCD4 induced gp120 shedding

In order to study the spontaneous shedding of gp120 from Env transfected 293T cells, cell-free supernatants were collected 48 hours post-transfection and filtered through a sterile 0.22 micron membrane. Then gp120 proteins present in the supernatant was purified by precipitation with lectin-agarose (Sigma-Aldrich) after overnight incubation in a rotator at 4°C and washed thrice with PBS. The washed agarose-beads were resuspended in 1X SDS-sample loading buffer, heated at 100°C and analyzed by Western blot using HIVIG human antibodies as probes. For deglycosylation of Env proteins, the precipitated and washed agarose-beads were resuspended in 1X deglycosylation buffer (G5 reaction buffer, New England Biolabs), digested with 500U of Endo H and 50U of Neuraminidase (New England Biolabs) overnight at 37°C and then analyzed by Western blot using HIVIG as probes.

Soluble CD4 induced shedding of gp120 was measured by FACS and ELISA. For FACS analysis, Env transfected 293T cells were harvested 48 hours post-transfection, washed extensively with FACS buffer, resuspended in 1ml of FACS buffer (12–20 X 10^6^ cells per reaction) and incubated in the absence or presence of sCD4 (50 g) for 1 hour at 4°C with intermittent mixing. The cells were centrifuged and the pellet was resuspended in FACS buffer and then added in a round-bottomed 96-well tissue culture plate (2.5 X 10^5^ cells/well) for staining with the indicated antibodies (VRC01, PGT121, b6, 7B2) for 1hour. The cells were then washed extensively with FACS buffer and incubated with PE-conjugated goat anti-human secondary antibody for 1hour. Finally, the cells were washed, resuspended in 0.5% paraformaldehyde (in PBS) and analyzed with a FACS Verse/Canto (BD Biosciences). Presence of gp120 in the supernatant of sCD4-treated or control cells were also measured by ELISA assay. Briefly, 100 μl of serially diluted (1:3) cell supernatants were added to a flat-bottomed 96-well plate that was previously coated overnight with 2 μg/ml lectin (Sigma-Aldrich) and pre-incubated with blocking buffer (5% milk, 5% HIFBS in PBS). Following three times washing with wash buffer (0.1% Tween-20 in PBS), anti-gp120 clade C rabbit polyclonal antibody (1:1000 dilution, Immunetech, USA) was added to the wells. The cells were again washed three times with wash buffer and then incubated with HRP-conjugated goat anti-rabbit secondary antibody (1:1000 dilutions, Santa Cruz Biotechnology). Following washing three times with wash buffer, 100 μl of TMB substrate (Pierce) was added to the wells. After 15 min., the reaction was stopped with 1N HCl and the absorbance was measured at 450 nm with a spectrophotometer (Beckman Coulter).

### Pseudovirus production and neutralization assay

Viruses pseudotyped with 4-2.J41 Env were produced by co-transfection of envelope expressing plasmid (pSVIII-env) with env-deleted HIV-1 backbone plasmid (pSG3ΔEnv) into 293T cells in 6-well tissue culture plates using FuGENE6 Transfection Reagent (Promega Inc). Cell supernatants containing pseudotyped viruses were harvested 48 hours post-transfection and then stored at -80°C until further use. The infectivity assays were done in Tzm-bl cells (1 X 10^5^ cells/ml) containing DEAE-Dextran (25 μg/ml) in 96-well microtiter plates and infectivity titers were determined by measuring luciferase activity using Britelite luciferase substrate (Perkin Elmer) with a Victor X2 Luminometer (Perkin Elmer).

Viruses pseudotyped with 4-2.J41 Env were assessed for their neutralization sensitivity against neutralizing and non-neutralizing antibodies in a Tzm-bl neutralization assay. Briefly, pseudoviruses (1 X 10^5^ RLU/well) were incubated with serial dilutions (2-fold) of monoclonal antibodies in duplicate wells of a 96-well flat-bottom culture plate. After 1 hour of incubation at 37°C, 1 X 10^5^ TZM-bl cells containing 25 μg/ml DEAE-Dextran were added to each well. After 48 hours, luciferase activity was measured using the Britelite luciferase substrate (Perkin Elmer). The IC_50_ values were defined as antibody concentrations that caused a 50% reduction in RLU compared to the virus control.

## Supporting Information

S1 Fig FACS-based cell surface binding curves of sCD4 treated or untreated 4-2.J41 Env expressing 293T cells. Mean fluorescence intensities (MFI) of binding of gp120-directed (VRC01, b6, PGT121) and gp41-directed antibodies (7B2) are shown. The graphs shown here are derived from the same representative experiments. Bars at each antibody concentration indicate the SEM values for duplicate samples.
(TIF)

S2 Fig Increased cell surface expression of tail-truncated and codon optimized 4-2.J41 Env. (A-B) Cell surface binding curves of wild-type and tail-truncated (delCT) or codon-optimized (CO) 4-2.J41 Env to PGT121 antibody. The graphs shown here are derived from the same representative experiments. Bars at each antibody concentration indicate the SEM values for duplicate samples.
(TIF)

S1 Table Ratio of binding to neutralizing (VRC01) versus non-neutralizing (F105) antibodies by different Indian clade C Envs in cell-surface binding assay. (DOCX)

S2 Table Average neutralization IC_50_ values of antibodies for 4-2.J41 and 4-2.J41delCT Env-pseudotyped viruses. (DOCX)
