# Supplementary figures and images for: An Efficiently Cleaved HIV-1 Clade C Env Selectively Binds to Neutralizing Antibodies
Source: PLoS One. 2015 Mar 30;10(3):e0122443. doi: 10.1371/journal.pone.0122443 (PMC4379091; doi:10.1371/journal.pone.0122443)

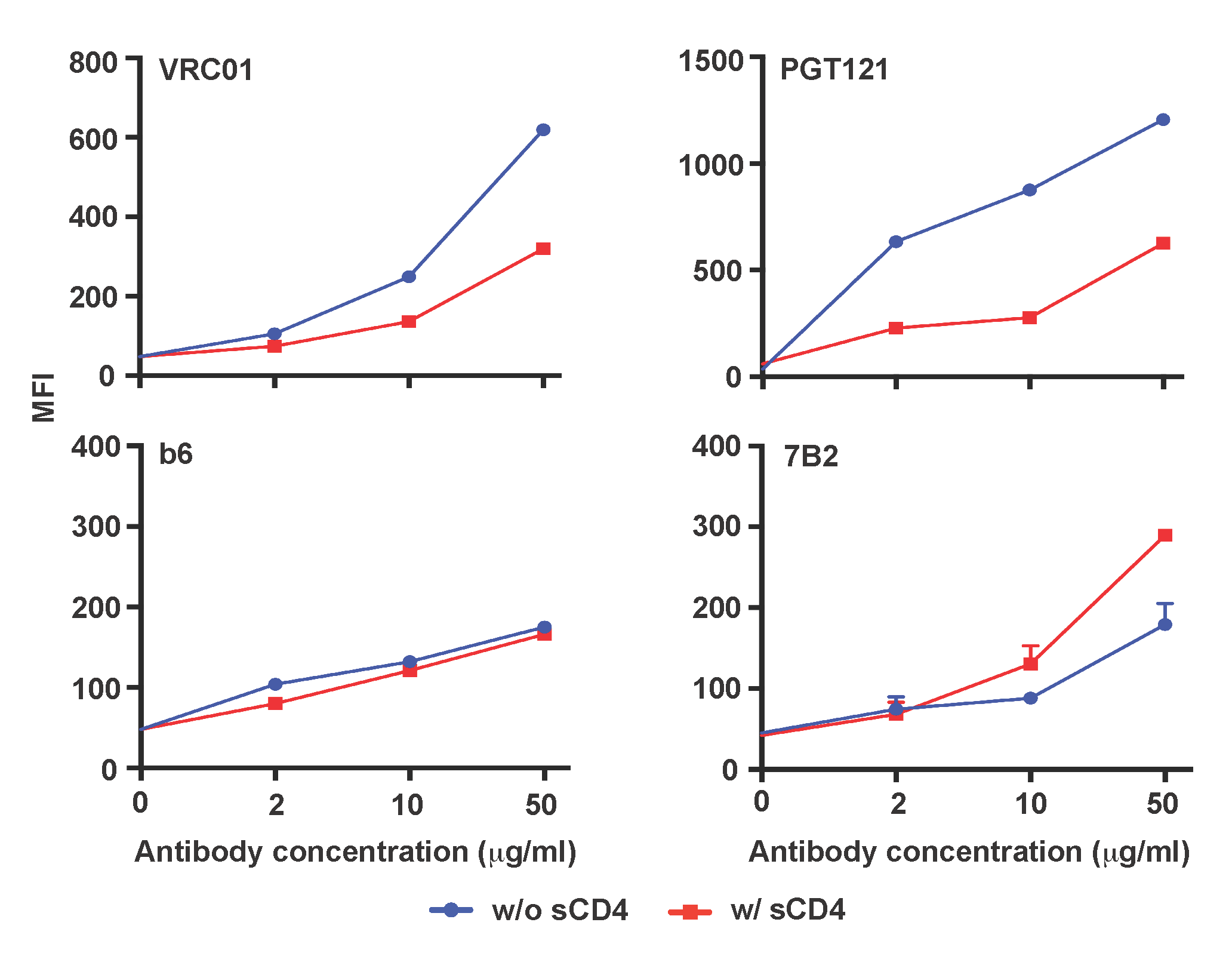

Supplement: S1 Fig — Mean fluorescence intensities (MFI) of binding of gp120-directed (VRC01, b6, PGT121) and gp41-directed antibodies (7B2) are shown. The graphs shown here are derived from the same representative experiments. Bars at each antibody concentration indicate the SEM values for duplicate samples. (TIF) [file pone.0122443.s001.tif]

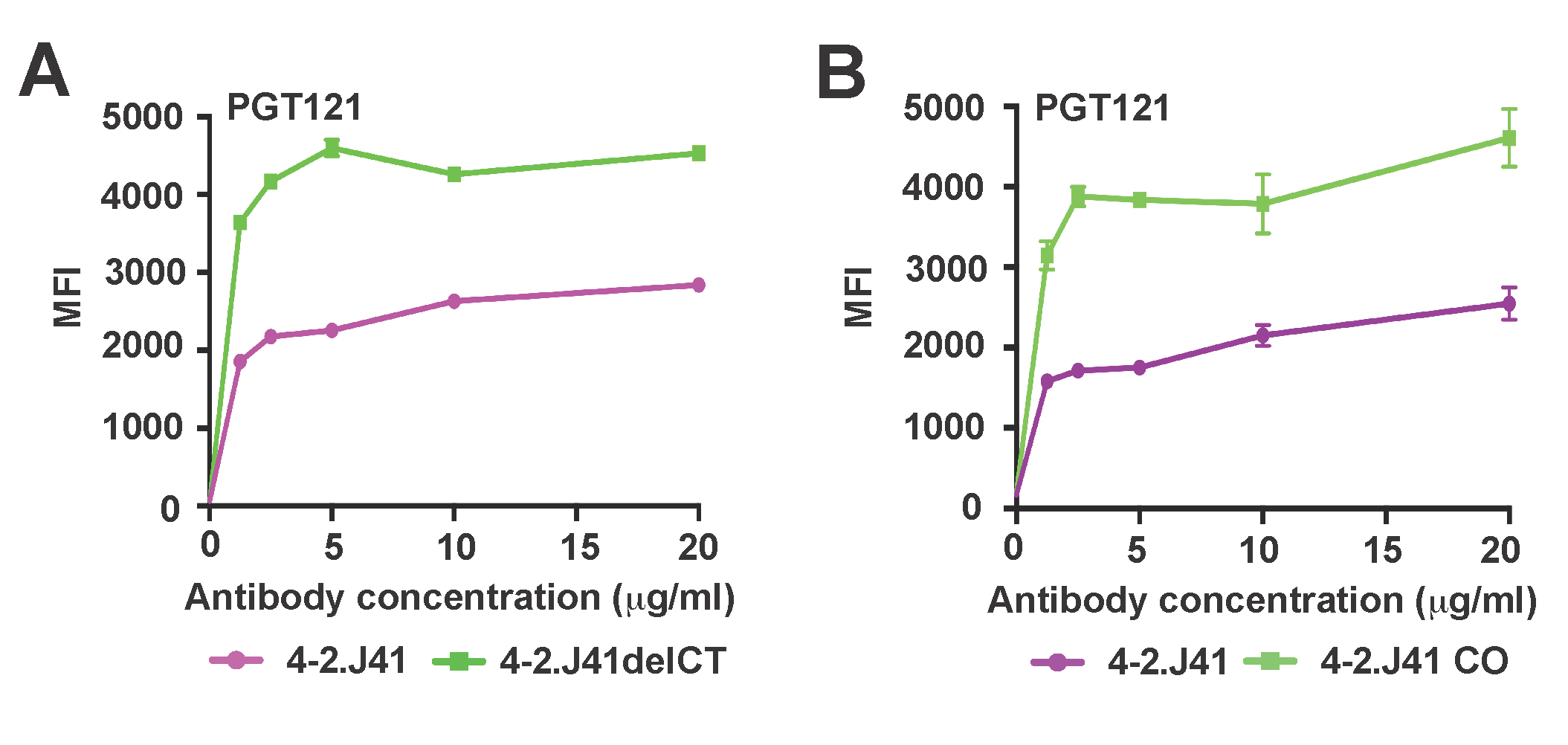

Supplement: S2 Fig — (A-B) Cell surface binding curves of wild-type and tail-truncated (delCT) or codon-optimized (CO) 4-2.J41 Env to PGT121 antibody. The graphs shown here are derived from the same representative experiments. Bars at each antibody concentration indicate the SEM values for duplicate samples. (TIF) [file pone.0122443.s002.tif]
