# Supplementary material for: An Efficiently Cleaved HIV-1 Clade C Env Selectively Binds to Neutralizing Antibodies
Source: PLoS One. 2015 Mar 30;10(3):e0122443. doi: 10.1371/journal.pone.0122443 (PMC4379091; doi:10.1371/journal.pone.0122443)
Supplement: S1 Table — (DOCX) [file pone.0122443.s003.docx]

**S1 Table:** Ratio of binding to neutralizing (VRC01) versus non-neutralizing (F105) antibodies by different Indian clade C Envs in cell-surface binding assay.

| HIV-1 Env Clone | Binding ratio (VRC01:F105) |
| --- | --- |
| 4-2.J41 | 4.7 |
| 11-3.J3 | 4.1 |
| 4-2.J42b | 3.6 |
| 3-5.J38 | 2.5 |
| 3.5.J25 | 2.5 |
| 2-7.J1 | 1.8 |
| 2-5.J11 | 1.7 |
| 2-11.J16 | 1.7 |
| LT1_09.J8 | 1.7 |
| LT1_09.J3 | 1.5 |
