# Supplementary material for: An Efficiently Cleaved HIV-1 Clade C Env Selectively Binds to Neutralizing Antibodies
Source: PLoS One. 2015 Mar 30;10(3):e0122443. doi: 10.1371/journal.pone.0122443 (PMC4379091; doi:10.1371/journal.pone.0122443)
Supplement: S2 Table — (DOCX) [file pone.0122443.s004.docx]

**S2 Table:** Average neutralization IC_50_ values of antibodies for 4-2.J41 and 4-2.J41delCT Env-pseudotyped viruses.

| Antibody | IC_50_(μg/ml) | |
| --- | --- | --- |
|  | 4-2.J41 | 4-2.J41delCT |
|  |  |  |
| VRC01 | 0.47 | 0.97 |
| PG9 | 0.54 | 2.92 |
| PGT121 | <0.02 | <0.02 |
| PGT145 | 6.69 | >10 |
| PGT151 | 0.03 | 0.23 |
| 4E10 | 2.14 | 9.34 |
| 10E8 | 2.16 | 2.24 |
| F105 | >50 | >50 |
| 17b | >50 | >50 |
| b6 | >50 | >50 |
| 7B2 | >50 | >50 |
